# RNAPII response to transcription‐blocking DNA lesions in mammalian cells

**DOI:** 10.1111/febs.16561

**Published:** 2022-07-04

**Authors:** Jianming Wang, Martina Muste Sadurni, Marco Saponaro

**Affiliations:** ^1^ Transcription Associated Genome Instability Laboratory, Institute of Cancer and Genomic Sciences University of Birmingham UK

**Keywords:** DNA damage, double strand break, homologous recombination, nucleotide excision repair, R‐loop, RNA polymerase II, RNA transcription

## Abstract

RNA polymerase II moves along genes to decode genetic information stored in the mammalian genome into messenger RNA and different forms of non‐coding RNA. However, the transcription process is frequently challenged by DNA lesions caused by exogenous and endogenous insults, among which helix‐distorting DNA lesions and double‐stranded DNA breaks are particularly harmful for cell survival. In response to such DNA damage, RNA polymerase II transcription is regulated both locally and globally by multi‐layer mechanisms, whereas transcription‐blocking lesions are repaired before transcription can recover. Failure in DNA damage repair will cause genome instability and cell death. Although recent studies have expanded our understanding of RNA polymerase II regulation confronting DNA lesions, it is still not always clear what the direct contribution of RNA polymerase II is in the DNA damage repair processes. In this review, we focus on how RNA polymerase II and transcription are both repressed by transcription stalling lesions such as DNA‐adducts and double strand breaks, as well as how they are actively regulated to support the cellular response to DNA damage and favour the repair of lesions.

## Graphical abstract

The RNA polymerase II transcription progression is affected by the presence of DNA damage events, such as DNA double strand breaks and UV‐induced lesions. It has long been considered that cells simply repress transcription to repair DNA damage. However, it has become clear that RNA polymerase II participates directly in the DNA damage response, recruiting DNA damage repair factors and supporting the repair through the production of specific RNA molecules.

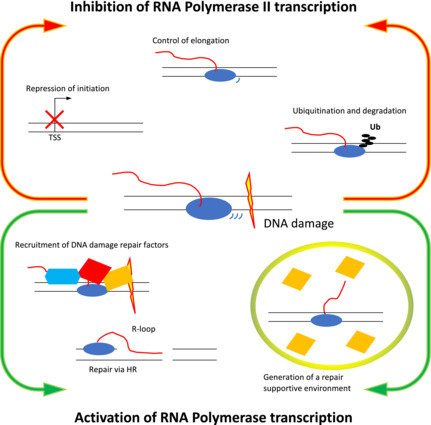

**Abbreviations**
6‐4PP: pyrimidine‐(6‐4)‐pyrimidone photoproduct
CPD: cyclobutane pyrimidine dimer
CSA: Cockayne syndrome A
CSB: Cockayne syndrome B
DDR: DNA‐damage response
dilncRNA: DSB‐induced long non‐coding RNA
DSB: double‐stranded DNA break
GG‐NER: global genome nucleotide excision repair
HR: homologous recombination
IEG: immediate early gene
NELF‐E: negative elongation factor E
NER: nucleotide excision repair
NHEJ: non‐homologous end joining
RNAPII: RNA polymerase II
TC‐NER: transcription coupled nucleotide excision repair

## Introduction

The genome integrity in mammalian cells is constantly threatened by endogenous and environmental agents, which can block DNA replication progression, as well as the progression of transcription [1]. To counteract the impact of DNA lesions, cells have evolved a comprehensive DNA‐damage response (DDR) network that regulates at different levels all cellular processes, including RNA polymerase II (RNAPII) transcription [1, 2]. Although RNAPII can bypass some types of DNA lesions such as some types of oxidative DNA damage [3], it is strongly affected by double‐stranded DNA breaks (DSBs) or bulkier lesions (Table 1).

**Table 1:** Transcription‐dependent DNA damage repair pathways dealing with specific DNA lesions.

Type of DNA damage	Transcription‐dependent repair pathways	References
Double strand break	HR	[4]
Single strand break	HR (not yet fully proved)	[5, 6]
UV‐induced intra‐strand crosslinks	NER	[7, 8]
Platinum/psoralen/formaldehyde crosslinks	NER	[9, 10, 11]
Protein crosslinks	NER	[12, 13]

Consequently, there are many DNA lesions that can inhibit transcription and, for RNAPII to resume its activity, these lesions must be repaired. Depending on the DNA damage repair kinetics of the different lesion types, RNAPII transcription can be affected for long time. For example, in the case of UV treatments, DNA damage will trigger a general shutdown of transcription with transcription activity returning to normal levels only after many hours [14]. However, in Cockayne syndrome patient cells that are defective with respect to the repair of UV‐induced DNA damage, transcription will not return to normal levels [14]. UV‐induced DNA lesions are repaired mainly by nucleotide excision repair (NER), whereas DSBs are repaired by non‐homologous end joining (NHEJ) and homologous recombination (HR) [7, 14, 15, 16, 17, 18]. Hence, in this review, we summarise how the RNAPII complex and RNAPII transcription are regulated, as well as directly regulate these processes. We also analyse the impact of endogenous sources of DNA damage that can negatively or positively affect RNAPII transcription, with their potential implications for human disease [11, 19].

## Transition coupled nucleotide excision repair (TC‐NER) and global genome nucleotide excision repair (GG‐NER)

UV light causes two major types of DNA lesions: cyclobutane pyrimidine dimers (CPDs) and pyrimidine‐(6‐4)‐pyrimidone photoproducts (6‐4PPs) [20]. If not repaired properly, these DNA lesions can interfere with DNA replication and transcription and lead to disease onset [20, 21]. It has been well known for decades that 6‐4PP photoproducts are removed faster than cyclobutane pyrimidine dimers [22]. UV‐induced photolesions can be repaired by NER, which is highly conserved in eukaryotes and is responsible for the removal of a broad range of DNA damage, including also bulky DNA adducts and DNA crosslinks [23]. NER can be categorised into two sub‐pathways: GG‐NER and TC‐NER. Both GG‐NER and TC‐NER can remove CPDs and 6‐4PPs in mammalian cells, with GG‐NER being important for fixing DNA lesion independent of transcription status, whereas TC‐NER repairs bulky lesions in the transcribed DNA strand [24]. This is because the two sub‐pathways differ with respect to their activation mechanisms, with TC‐NER being activated directly by RNAPII stalled at a DNA damage site; GG‐NER instead removes DNA lesions at any location in the genome activated by the distortion of the DNA helix induced by the lesion [20]. Following their activation, both GG‐NER and TC‐NER use the same cascade of enzymes to process and remove the damaged DNA [20]. Nevertheless, by comparing chromatin features and kinetics of DNA excision repair globally in the human genome, it appears that both repair pathways selectively remove preferentially DNA damage in active and open chromatin, with the persistence of damage in heterochromatin being linked to increased mutagenesis [25].

## TC‐NER factors

**Fig. 1:**
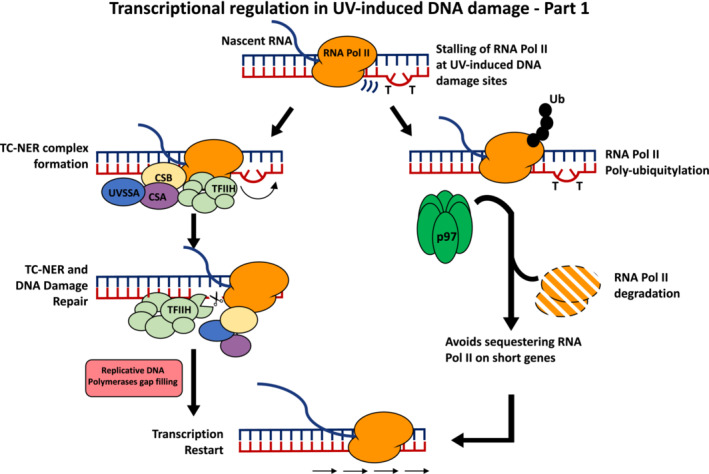
Treatment with UV light induces DNA damage such as pyrimidine dimers, which impair RNAPII progression along genes. Stalling of the RNAPII at the DNA damage site triggers the recruitment of TC‐NER factors, among which CSB, CSA and UVSSA play pivotal roles in the organisation of a functional TC‐NER complex. This complex will push forward RNAPII to get access to the damage site and repair it allowing eventually the RNAPII to continue its task. In parallel, TC‐NER is important for the ubiquitination and degradation of RNAPII, a step required for a functional shutdown of transcription, limiting the transcription of short genes that can sequester away RNAPII from longer genes, in this way impairing the restart of transcription.

TC‐NER was firstly discovered in mammalian cells [15, 16, 17, 26]. Subsequently, several TC‐NER factors have been identified using a multitude of approaches [23]. Cockayne syndrome A and B (CSA and CSB) proteins, for which mutations lead to Cockayne syndrome, are responsible for the first stages of TC‐NER and were first shown to be required in response to UV damage by Van Gool *et al*. [27]. CSB stably associates with RNAPII and also has crucial roles in transcription besides a role following UV‐induced DNA damage [27, 28, 29, 30], even though the interaction between RNAPII and CSB increases following UV damage [31]. Stalling of RNAPII at UV‐induced DNA damage comprises the signal that leads to the recruitment of CSB at sites of DNA damage and the activation of TC‐NER (Fig. 1). Importantly, the transcription shutdown following UV DNA damage is not the only consequence of the presence of unrepaired DNA damage that can affect transcription progression because undamaged DNA will similarly not be transcribed following UV‐induced DNA damage [32]. At the same time, cells reprogram their transcription profiles following UV DNA damage inducing the transcription of thousands of genes, with a preference for short genes [33, 34]. All of this indicates that the cellular regulation of RNAPII transcription in response to UV DNA damage is an active process that is established in affected cells, with CSB being required later on for transcription restart [32].

CSB is a SWI/SNF‐like ATPase for which its activity is required for stable association with chromatin after UV treatment [20, 35]. Sequence analysis identified a ubiquitin‐binding domain located in the C‐terminal of CSB, which is essential for TC‐NER in human cells [36]. A CSB mutant that lacks the ubiquitin‐binding domain can still support the assembly of the TC‐NER complex at UV damaged DNA sites but fails to repair the damage [36]. CSB is highly modified by several post‐translational modifications such as SUMO (i.e. small ubiquitin‐like modifier), ubiquitination and phosphorylation, although not all of these modifications control its roles exclusively in TC‐NER [37, 38, 39]. A detailed understanding of how CSB interacts with an UV‐arrested elongating RNAPII can be obtained by structural studies of CSB and its yeast homologue Rad26 [40, 41, 42]. What these studies have shown is that, following the arrest of the RNAPII, there is a reorganisation of the transcription elongation complex, with the replacement of the pro‐elongation complex DSIF by the repair complex formed by CSA‐CSB‐DDB1 [42]. CSB and its homologue Rad26 bind directly to RNAPII and DNA upstream of RNAPII, using their translocase activity to push the RNAPII forwards to bypass the lesion and making the DNA damage accessible to other components of the TC‐NER and enabling repair [41, 42]. In case the lesion cannot be bypassed, the CSA‐CSB‐DDB1 complex will establish Cullin‐ring E3 ubiquitin ligase recruiting Cullin4A and ROC1/Rbx1, where CSA acts as a substrate‐specifying subunit, to ubiquitylate and degrade RNAPII [20, 23, 42] (Fig. 1).

Apart from RNAPII, another target of the E3 ligase activity of the CSA‐associated complex is CSB itself, which is targeted for ubiquitylation and degradation, a step required for transcription recovery [43]. Upon UV damage, CSA is enriched in the nuclear matrix in a CSB‐dependent manner, colocalising with hyperphosphorylated RNAPII, and is important for the recruitment and establishment of a complete TC‐NER complex also involving TFIIH and UVSSA [42, 44, 45]. TFIIH is a multi‐subunit protein complex that also contains the XPB and XPD DNA‐dependent helicases, mutated in the genetic syndrome Xeroderma pigmentosa, which are responsible for the opening and unwinding of the duplex DNA at the site of damage [20, 23, 46]. UVSSA was initially identified to be important for promotomg stabilisation of CSB and supporting transcription recovery after UV damage, although more recent data show that UVSSA is important for the formation and establishment of a stable TC‐NER complex associated with RNAPII [42, 45, 47, 48, 49]. More recently, additional TC‐NER factors have been identified as being crucial in the regulation of the initial steps of the process. CSA interacts with the TriC chaperonin complex, and this is important for preserving CSA stability in cells [50]. ELOF1 has been identified as a transcription elongation factor that is important for supporting TC‐NER following UV damage, promoting the recruitment of UVSSA and TFIIH at DNA damage sites and RNAPII ubiquitylation [51, 52, 53]. Finally, following the formation of a mature TC‐NER complex, a pre‐incision complex is assembled around TFIIH to perform a dual incision at the damage site using the nucleases XPG and ERCC1‐XPF, removing the lesion containing DNA [20, 22]. The single‐stranded DNA gap is filled by the replicative DNA polymerases delta or epsilon and the nick is sealed [20, 21, 22]. Following the repair of the DNA damage, RNAPII can resume and complete the transcription process.

## RNAPII ubiquitylation and degradation

As mentioned above, one of the targets of the ubiquitylation processes at sites of UV‐induced DNA damage is the RNAPII itself. Indeed, for long time, it was assumed that the backup pathway for TC‐NER following UV‐induced DNA damage in case lesions that cannot be accessed by pushing forward RNAPII involves freeing the DNA damage site for its repair through the ubiquitylation and degradation of the largest subunit of the RNAPII complex, RPB1 [53]. Because of its destructive impact, ubiquitylation and degradation of RPB1 is a highly regulated and finely tuned process. RPB1 ubiquitylation goes through two stages, with an initial monoubiquitylation performed by the E3 ubiquitin ligase NEDD4 [54], and a poly‐ubiquitylation performed by a complex formed by Elongin‐RBX1‐Cullin [55]. VCP/p97 is additionally required following UV‐induced DNA damage for both proteasome‐mediated RNAPII degradation in mammalian cells [56] and CSB degradation [57].

Nevertheless, more recently, the ubiquitylation of RPB1 has been shown to be an essential step in the regulation of UV‐induced DNA damage [58, 59]. Nakazawa *et al*. [58] reported that RPB1 ubiquitylation at K1268 is required for the interaction between the TFIIH core complex and UV‐stalled RNAPII, and that ubiquitylation of UVSSA at K414 mediates such an interaction. Moreover, ubiquitylation of RNAPII at RPB1‐K1268 plays an important role in the appropriate and efficient repair of UV‐induced DNA damage and in preventing the development of premature ageing and neurodegeneration in mice [58]. In parallel, Tufegdzic Vidakovic *et al*. [59] report that RPB1‐K1268 is essential for RNAPII polyubiquitylation and degradation following UV damage, being important for appropriate DNA damage repair kinetics and survival following UV treatment. Moreover, ubiquitylation of RPB1 at K1268 is required for the correct shutdown of transcription initiation following UV damage and for transcription recovery in CSB defective cells [59]. Altogether, in recent years, the interpretation of RNAPII ubiquitylation has changed from an undesired consequence of UV damage to a necessary step in the repair of UV‐induced DNA damage (Fig. 1). Defective RNAPII degradation leads to an accumulation and sequestering of RNAPII on short genes for which transcription is maintained following UV‐induced DNA damage, affecting the ability to reset the transcription program [58, 59].

## Global transcriptional response to UV damage

**Fig. 2:**
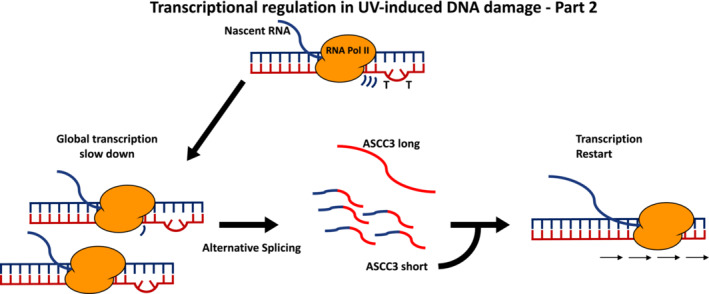
Following UV‐induced DNA damage, cells will slow down transcription elongation and shutdown transcription initiation, affecting most of the genes independently of the presence of DNA lesions. The slowdown of transcription elongation induces a series of alternative splicing events, among which there is a high incidence of inclusions of alternative last exons, which leads to the production of shorter transcripts. In a specific case, the inclusion of an alternative last exon in the protein coding gene ASCC3 forms a small non‐coding RNA that is essential for transcription recovery, in a first case of a gene that can generate both a protein coding and a non‐coding RNA.

Equally, the mechanisms behind transcription shutdown following UV damage have recently been characterised in more detail. It has long been known that transcription is blocked by UV‐induced DNA lesions from prokaryotes to eukaryotes [60, 61]. In response to UV‐induced DNA lesions, transcription is temporarily repressed but later recovers in human cells, with cells deficient in TC‐NER being unable to restore RNA synthesis [14]. The reduction in transcription activity is associated with a strong decrease in the hypophosporylated form of RNAPII, indicating a repression of transcription initiation, with a stronger decrease in cells defective in TC‐NER components [62]. This indicates that UV treatment induces a block of transcription elongation because of the presence of lesions and a repression of transcription initiation, both of which require TC‐NER factors [32, 62, 63]. Investigation of the genome‐wide effects of UV‐irradiation on transcription and DNA damage repair kinetics reveals that UV‐exposure preferentially inhibits transcription elongation of longer genes, and that lesion removal of longer genes is faster from the 5′ ends than from the 3′ ends [64, 65, 66]. Indeed, it has been proposed that the reduction of transcription initiation events is also obtained by unleashing RNAPII from promoters to transcribe into genes and act as a sensor for DNA damage, supporting the repair of lesions [66]. Importantly, the transcription elongation slowdown is equally functionally relevant and important because it allows the generation by alternative splicing of a series of transcripts, among which a short form of the protein coding gene ASCC3 stands out that is generated by alternative splicing of an alternative last exon [64]. Intriguingly, this short ASCC3 mRNA is a non‐coding RNA that is specifically required to recover transcription activity after UV‐induced DNA damage [64] (Fig. 2).

Nevertheless, as previously mentioned, even with global transcription shutdown following UV‐induced DNA damage, many genes become actively transcribed. As in the case of ionising radiation treatments, UV‐induced DNA damage activates the transcription of a series of so called immediate early genes (IEG) [67, 68, 69]. A key factor involved in the cellular transcriptional response to UV damage is p53, for which activity is also important for the repair of UV‐induced DNA damage [70, 71, 72]. One feature of IEG, and in particular of p53‐induced genes, is that these genes tend to be relatively short [73]. Because UV‐induced DNA damage will start slowing down gene transcription after the first 20–30 kb from TSS [64], and because UV‐induced DNA damage will be repaired faster for shorter genes than longer genes [65, 66], an IEG can still be transcribed in the presence of DNA damage. However, after this initial cellular response to stress, RNAPII degradation is functional with respect to avoiding short genes sequestering RNAPII complexes and, in this way, limiting and restricting the possibility of re‐expressing longer genes, supporting a reset of the transcription program [59].

## RNAPII response to DSB

**Fig. 3:**
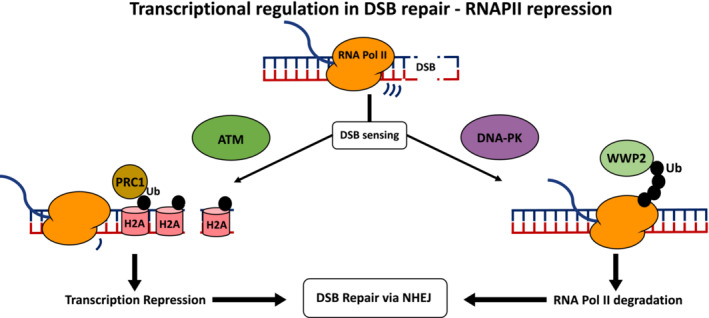
In the case of a DSB, RNAPII transcription will be repressed only in *cis* next to the DNA damage site. This is achieved via changes to the chromatin organisation, such as ubiquitination of H2A triggered by ATM to shut down gene transcription and RNAPII ubiquitination and degradation triggered by DNAPK, favouring the repair of the DSB by NHEJ.

By contrast to UV treatment, DSBs do not lead to a global shutdown of RNAPII transcription but, instead, they induce a shutdown of specifically RNA polymerase I transcription in an ATM‐dependent manner [74]. RNAPII transcription is affected locally, however, when the affected gene is in proximity of the damage DNA site [75, 76]. The transcriptional silencing is coordinated by ATM‐dependent local chromatin modifications, through histone H2A ubiquitylation and inhibition of RNAPII elongation, even though there is a concomitant inhibition of transcription initiation at genes that contain a DSB [75, 77]. Several ATM targets have already been identified, such as the chromatin remodelling complex PBAF, which is important for the transcriptional silencing and the repair of DSBs [78]. During S phase, ATM and CDK2 regulate CSB recruitment to DSBs to remodel chromatin, in this way limiting the accumulation of RIF1s at these sites in favour of BRCA1 [79]. ATM regulates the transcription elongation factor ENL by phosphorylation increasing its interaction with the E3 ubiquitin ligase PRC1, leading to H2A ubiquitylation [80]. More recently, it has been shown that DNA‐PK_CS_ and the ubiquitin ligase WWP2 are important for regulating RNAPII levels at sites of DSBs and its degradation to support channelling the repair into NHEJ [4, 81] (Fig. 3).

However, the list of factors involved in RNAPII regulation at DSBs is much more extensive. The negative elongation factor E (NELF‐E) is involved in the inhibition of local RNAPII transcription downstream of site‐specific DSBs in mammalian cells [82]. Following DSB induction, NELF‐E is recruited to the DNA damage site by the presence of RNAPII and poly‐ADP‐ribose polymerase 1, to block active gene transcription and regulate DSB repair [82]. Cohesin is similarly required for the repression of transcription at sites of DSBs and is important for reducing the formation of rearrangements involving these sites [83].

## Positive role of RNA transcription in DSB repair

On the other hand, although there is clear evidence that RNAPII is inhibited at DSBs, there is equally clear evidence showing that transcription activity is required for DSB repair and is important for maintaining genome integrity. The first evidence of a role of transcription in DSB repair was the evidence that 53BP1 requires RNA for its association with the chromatin because treatment with RNase A, but not RNase H, affects 53BP1 DNA damage‐induced foci formation [84]. Importantly, the treatment with RNase A was able to affect all 53BP1 foci formation, suggesting that the presence of RNA at DSB sites could have a critical role for the correct assembly of 53BP1 foci globally, and was not only something specifically linked to DSBs in transcribed regions [84]. Indeed, small non‐coding RNAs are purposely produced at the DSB site through the DICER and DROSHA RNases involved in the RNA interference pathway, regulating DDR activation in a MRE11–RAD50–NBS1‐dependent manner in mammalian cells [85, 86] (Fig. 3). DDR foci formation is furthermore sensitive to both RNase A and a‐amanitin treatments [85]. The small non‐coding RNAs termed DNA damage response RNAs come from DSB induced long non‐coding RNAs (dilncRNAs), which are transcribed around DNA broken ends [85, 87]. The interaction between DNA damage response RNAs and their precursors dilncRNAs at the site of damage thus stimulates DDR foci formation and DNA damage repair, channelling the repair towards RAD51‐dependent homologous recombination [87, 88]. This process is highly conserved across evolution and also is present in *Saccharomyces cerevisiae* [89]. Although initial studies suggested that RNAPII was responsible for the production of dilncRNAs because of the α‐amanitin sensitivity, newer evidence suggests that RNA polymerases other than RNAPII are involved in the generation of non‐coding RNAs at DSB sites because RNAPIII that is also α‐amanitin sensitive is recruited to these sites by MRE11 [90], whereas plants use their specialised RNA polymerase IV to generate non‐coding RNAs at DSBs [91]. RNA polymerases are found at DSBs together with transcription‐associated factors such as components of the transcription preinitiation complex, the MED1 part of the mediator complex and other transcription elongation factors, all in an MRN‐dependent manner and important for activating RNAPII transcription [92]. Moreover, the enrichment of RNAPII at DSB is stimulated by the Abelson tyrosine kinase c‐Abl, which mainly phosphorylates a Tyr1 residue at the C‐terminal domain of RNAPII promoting the production of small double‐stranded RNA around DSB and the formation of DDR foci [93]. Importantly, the production of RNA at these sites is relevant for the generation of a phase separation [92]. Phase separation is a process that has recently attracted notable interest because it appears to be important for supporting the recruitment of DNA damage response factors at sites of damage and the establishment of a DNA damage response [94, 95].

**Fig. 4:**
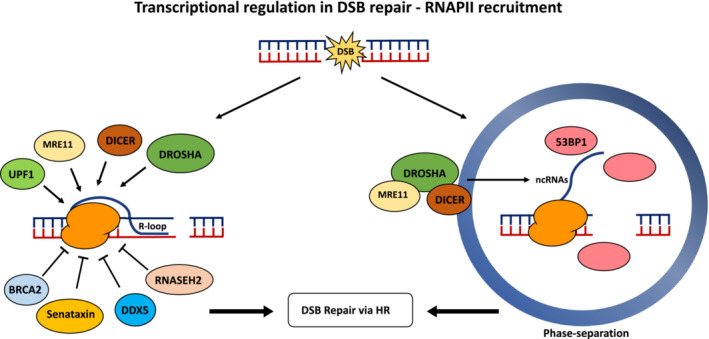
RNA polymerases can be loaded at sites of DSB to generate site‐specific non‐coding RNAs, in a process supported by several factors, including MRE11, DROSHA and DICER. These non‐coding RNAs have important structural roles in the establishment of appropriate 53BP1 foci and support the generation of a phase separation. In parallel, there is an intermediate generation of R‐loops at DSBs important for channelling the repair along a RAD51‐dependent HR DSB repair pathway, with R‐loop stability controlled by several proteins, including senataxin, RNASEH2 and BRCA2.

An important intermediate in the production of these dilncRNAs is the formation of RNA–DNA hybrids called R‐loops. Although R‐loops have long been associated with increased genome instability, in particular as a source of replication stress, at DSB sites, their role appears to be different [96]. RAD52 is important for the processing of R‐loops so that DSB can be repaired by RAD51‐dependent homologous recombination, in a process described as transcription associated‐homologous recombination repair [89, 97]. Appropriate processing of these R‐loops is an essential part of the DSB repair process because defects in R‐loop processing will induce increased genome instability [89, 98]. Indeed, several factors such as senataxin, CSB, EXOSC10 and BRCA2, through the recruitment of RNASEH2 and DDX5, are important for R‐loop processing, aiming to avoid DNA damage persistence and the incorrect repair of DNA lesions [99, 100, 101, 102, 103, 104]. In parallel, DROSHA, RBM14 and UPF1 are important for the establishment and formation of R‐loops at DSB sites [105, 106, 107]. Equally, much research has indicated that dilncRNAs are heavily modified and edited, and, in particular, methylation of the RNA is important for the correct processing of R‐loops and promoting homologous recombination [108, 109, 110, 111] (Fig. 4).

## Endogenous sources of transcription stalling lesions and their impact on transcription

Even though the above‐mentioned pathways have been shown to be important for the repair of exogenous lesions, recent evidence highlights how the DNA damage response pathways have evolved to counteract endogenous sources of DNA damage. For example, recent data has shown that TC‐NER factors are important with respect to dealing with formaldehyde‐induced DNA damage, a reactive intermediate and by‐product of many metabolic pathways [11]. Intriguingly, this parallels what is observed in mice models of TC‐NER mutants, where the lifespan of these mice can be extended by dietary restriction, in a process that ultimately reduces endogenous DNA damage levels by reducing the level of reactive species produced by metabolic processes [112].

Similarly, endogenous DSBs can occur in cells in at least two conditions and RNAPII transcription is directly involved in the process: class‐switch recombination during antibody generation and hormone‐dependent transcription‐induced DSB [19, 113, 114]. In the first case, RNAPII is instrumental for the process because the production of a transcript will lead to the formation of R‐loops in specific regions along the gene, which triggers AID activity and induces a DSB required to perform the class‐switch [115]. In the second case, oestrogen‐ and androgen‐stimulated gene activation is dependent upon the recruitment of Top2 on the gene promoter, inducing the formation of a DSB to trigger gene expression [19, 116]. In both cases, if DSBs are not repaired correctly, this can lead to the formation of chromosomal rearrangements [115, 116]. However, unexpectdly, even though Top2‐induced DSB induce a DNA damage response with activation of ATM and formation of gH2AX foci, transcription is not repressed as previously described [75, 76, 77, 78, 79], but will be activated [116].

## Conclusions and perspective

Altogether, 30 years of research on UV‐induced DNA damage has demonstrated how RNAPII transcription is dramatically affected by the treatment, with huge impacts on the ability to transcribe genes and the stability of the RNAPII complex itself. Nevertheless, these studies have also shown that the regulation of RNAPII protein stability itself plays a direct role in the repair of DNA damage and the recovery of transcription activity [58, 59, 64]. The list of factors involved in TC‐NER is perhaps not yet complete because functional and proteomic studies have identified many proteins that could have a role in this process [50, 64, 117]. Consequently, it should be expected that novel findings could be on the horizon, potentially allowing us to determine more mechanistic details regarding human diseases.

In the case of DSB repair, our understanding on how RNAPII is regulated by the damage and the relevance of RNA transcription in the repair of the lesions has vastly changed over recent years. Although initial findings showed only a localised removal and degradation at DSB sites [75], more recently, the active loading of RNA polymerases has been shown to be similarly important for channelling the repair towards the HR pathway [85, 97]. It is perhaps this that comprises a key role for transcription at sites of DNA damage because actively transcribed regions are preferentially repaired by HR, whereas not transcribed regions are repaired by NHEJ [118]. This could be directly affected by the presence of RNAPII at sites of DNA damage, or by specific histone modifications present near the DSB site [18]. Perhaps it is possible to speculate that the specific chromatin environment present at promoters of oestrogen and androgen responsive genes could stop the DNA checkpoint from repressing gene transcription. On top of this, RNA polymerases could play additional roles in DNA damage repair because the RNA present at DSB sites could serve as a template for the actual repair process [119, 120, 121]. Future research will help to determine the overall contribution of the RNA produced at DSB sites with respect to supporting DNA damage repair and also clarify whether nascent transcripts produced on the undamaged allele can be generated specifically to support the repair of the lesion. These nascent transcripts represent faithful copies of the DNA content, although their usability for the DNA damage repair is limited by their very short life because splicing is co‐transcriptional with RNAPII progressing along the gene [122]. Finally, it will also be important to assess whether the presence of RNA polymerases, or of RNA transcripts, can play a role in the repair of other types of DNA damage, or in channelling the repair towards specific pathways.

## Conflict of interest

The authors declare that they have no conflicts of interest.

## Author contributions

JW wrote the paper. MMS prepared the figures in the paper. MS edited the paper and reviewed the final version submitted for publication.

## Data Availability

Data sharing not applicable – no new data generated.
